# Establishment of a repertoire of fertility associated sperm proteins and their differential abundance in buffalo bulls (*Bubalus bubalis*) with contrasting fertility

**DOI:** 10.1038/s41598-023-29529-5

**Published:** 2023-02-08

**Authors:** Arumugam Kumaresan, Manish Kumar Sinha, Nilendu Paul, Pradeep Nag, John Peter Ebenezer Samuel King, Rakesh Kumar, Tirtha Kumar Datta

**Affiliations:** 1 Theriogenology Laboratory, Southern Regional Station of ICAR-National Dairy Research Institute, Bengaluru, Karnataka 560030 India; 2 Animal Genomics Laboratory, ICAR-National Dairy Research Institute, Karnal, Haryana 132 001 India; 3 ICAR-Central Institute for Research on Buffaloes, Hisar, Haryana 125 001 India

**Keywords:** Biological techniques, Biotechnology, Physiology, Zoology

## Abstract

Sperm harbours a wide range of proteins regulating their functions and fertility. In the present study, we made an effort to characterize and quantify the proteome of buffalo bull spermatozoa, and to identify fertility associated sperm proteins through comparative proteomics. Using high-throughput mass spectrometry platform, we identified 1305 proteins from buffalo spermatozoa and found that these proteins were mostly enriched in glycolytic process, mitochondrial respiratory chain, tricarboxylic acid cycle, protein folding, spermatogenesis, sperm motility and sperm binding to zona pellucida (p < 7.74E−08) besides metabolic (p = 4.42E−31) and reactive oxygen species (p = 1.81E−30) pathways. Differential proteomic analysis revealed that 844 proteins were commonly expressed in spermatozoa from both the groups while 77 and 52 proteins were exclusively expressed in high- and low-fertile bulls, respectively. In low-fertile bulls, 75 proteins were significantly (p < 0.05) upregulated and 176 proteins were significantly (p < 0.05) downregulated; these proteins were highly enriched in mitochondrial respiratory chain complex I assembly (p = 2.63E−07) and flagellated sperm motility (p = 7.02E−05) processes besides oxidative phosphorylation pathway (p = 6.61E−15). The down regulated proteins in low-fertile bulls were involved in sperm motility, metabolism, sperm-egg recognition and fertilization. These variations in the sperm proteome could be used as potential markers for the selection of buffalo bulls for fertility.

## Introduction

The river buffalo (*Bubalus bubalis*), fondly called as “Black Gold”, is a unique dairy animal species in the Asia. Out of the 208 million buffaloes in the world, Asia alone possesses 201 million buffaloes (96.8% of the world buffalo population) contributing to 35.3% of the total milk production. In India, 49% of the total milk production is from the buffaloes. Besides the milk production, the contribution of buffaloes towards meat and draught is immense^1^. In spite of such huge contribution towards food and nutritional security of significant proportion of the human population, buffaloes are yet to receive due recognition in comparison to cattle. Although it is not wise to compare buffalos with cattle, often buffaloes are compared with cattle and reported to have higher incidence of subfertility/infertility problems. The buffalo has been traditionally regarded as a poor breeder because they require a greater number of inseminations for conception in spite of the conditions under which they are raised^2–4^. Among the several reasons, poor conception rate with artificial insemination is one of the major reasons limiting the exploitation of full production potential^5^. Both male and female contribute to conception, however, the role of males is amplified in artificial insemination because one bull is used to artificially bred several thousands of females. Buffalo spermatozoa are unique in terms of structural, compositional and functional attributes, and thus the fertility prediction methods/tools used in cattle may not hold good for this species^6,7^. Insemination of females using semen from low-fertile bulls causes significant loss to the dairy industry^8,9^. Therefore, it is imperative to find out some reliable tools/tests to determine the latent fertility of buffalo bulls so that the reproduction efficiency of this species can be improved.

Earlier, few studies have been conducted to find out sperm phenotypic differences between high- and low-fertile buffalo bulls and reported striking differences in sperm functional parameters between those bulls^6,10,11^. Similarly, studies conducted on buffalo bulls with different fertility ratings to find out the differences in the ability of spermatozoa to bind with oviduct indicated that the sperm-oviduct binding index was corelated with bull fertility^12,13^. However, their usefulness in buffalo bull fertility prediction is not consistent because sperm functional attributes and oviduct binding ability were influenced by external factors^14^; therefore precise estimation of buffalo sperm fertilizing potential still remains a challenge. Recently, it is well understood that besides the sperm phenotypic characteristics and functionalities, the molecular health of sperm plays a vital role in fertility and embryonic development. With the developments in analytical techniques and bioinformatics, the use of the novel ‘omics’ approach has emerged as a bright spot and open great scope for identifying sperm molecules associated with fertility, which can predict the latent fertility of bulls with great accuracy. Using sperm transcriptomic approach, very recently, the fertility associated transcripts have been identified in buffaloes^15^. This study reported that genes associated with oxidative phosphorylation pathway and embryonic development were significantly dysregulated in low-fertile compared to high-fertile buffalo bulls. This preliminary report on buffaloes indicate greater possibilities of identification of fertility markers using “omics” approach.

Since spermatozoa are terminally differentiated cells and lack the ability to synthesize new proteins, proteomic analysis may help to determine their accurate fertilizing ability. Therefore, proteomics approach has been widely accepted as the primary choice for identifying fertility biomarkers in sperm and seminal plasma of humans and animals^16^. Spermatozoa interact with several proteins during various phases of their lifecycle starting from spermatogenesis to ejaculation, which are essential for acquiring fertilization potential. Any defects or altered expression of proteins at any of these stages may lead to fertilization failure. Therefore, proteomic studies would be beneficial to decipher the roles of sperm proteins on fertility. During the last decade, the proteomic approach helped to find out answers for many of hitherto unanswered questions related to sperm function and bull fertility^17–20^. Very recently, a preliminary study conducted using 2D-DIGE approach reported that sperm proteins associated with energy metabolism and capacitation were differentially expressed between high- and low-fertile buffalo bulls^21^. However, information on global proteomic profile of buffalo spermatozoa and differences in global proteomic profile of spermatozoa between high- and low-fertile buffalo bulls is very limited. In spite of the fact that proteins are important for sperm functions and fertility, the possible sperm proteins controlling the fertility in buffaloes were barely addressed.

We hypothesised that protein expression in spermatozoa differ between high- and low-fertile buffalo bulls and deciphering this difference will help us to identify fertility biomarkers. Therefore, the aim of the current study was (i) to extensively characterize buffalo bull sperm proteins and (ii) to identify the fertility associated proteins in buffalo bulls. We used high-throughput liquid chromatography–mass spectrometry (LC–MS)-/MS-based differential proteomics approach to understand the alterations in the proteomic profile between high- and low-fertile buffalo bulls.

## Results

### Global proteomic profile of buffalo spermatozoa

**Figure 1:**
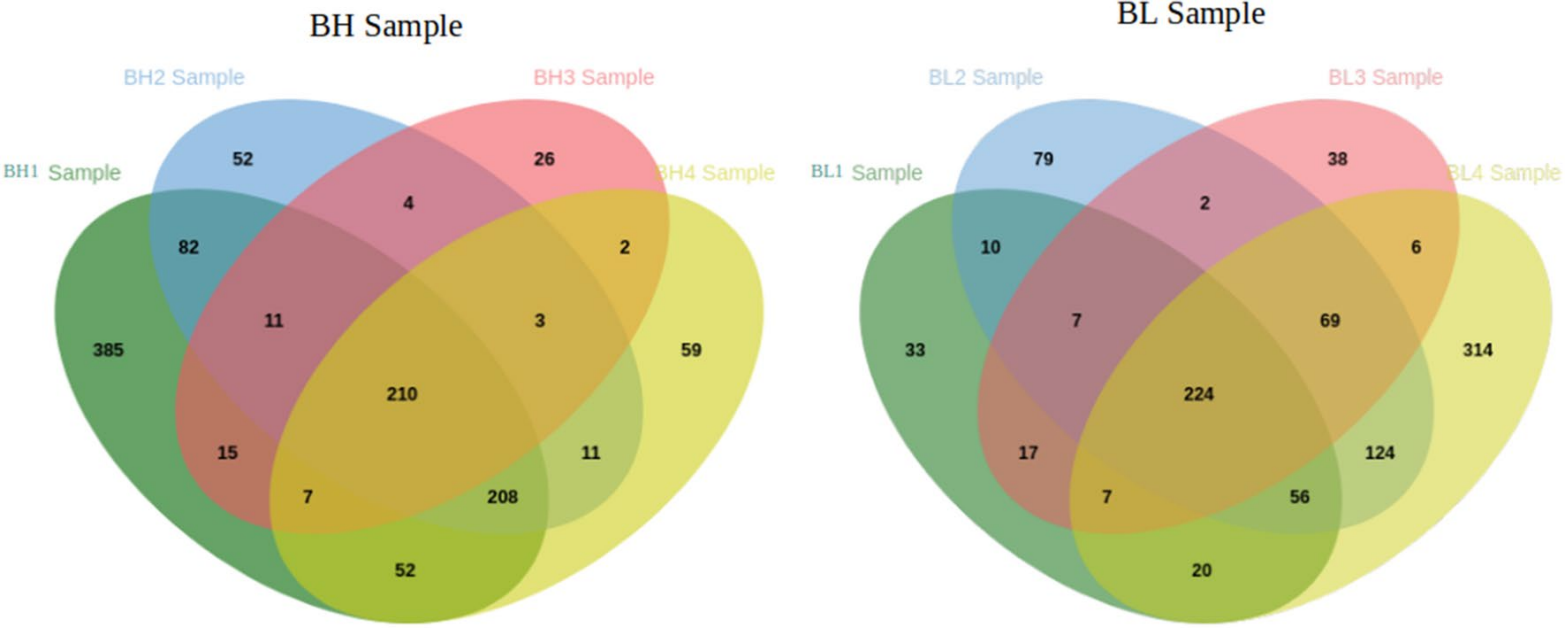
Venn diagram representing total number of proteins detected in buffalo bull spermatozoa. *BH* High-fertile buffalo bulls (n = 4), *BL* Low-fertile buffalo bulls (n = 4).

**Figure 2:**
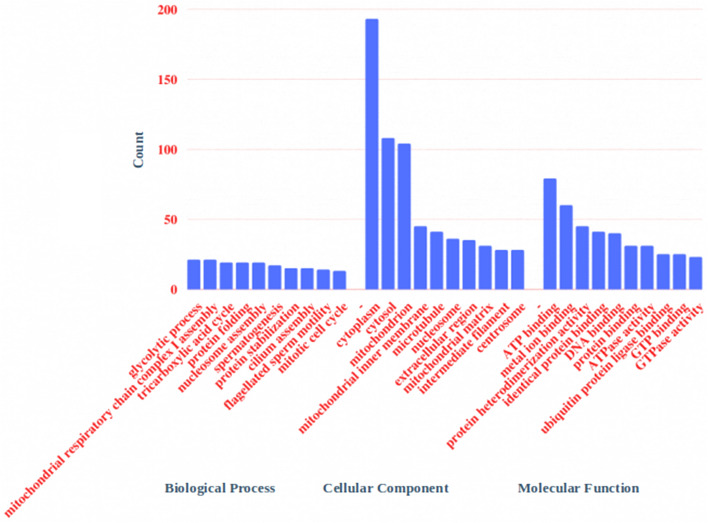
Gene ontology of global proteome of buffalo bull spermatozoa. Top 10 GO terms (biological process, cellular component and molecular function) are indicated in the figure.

**Figure 3:**
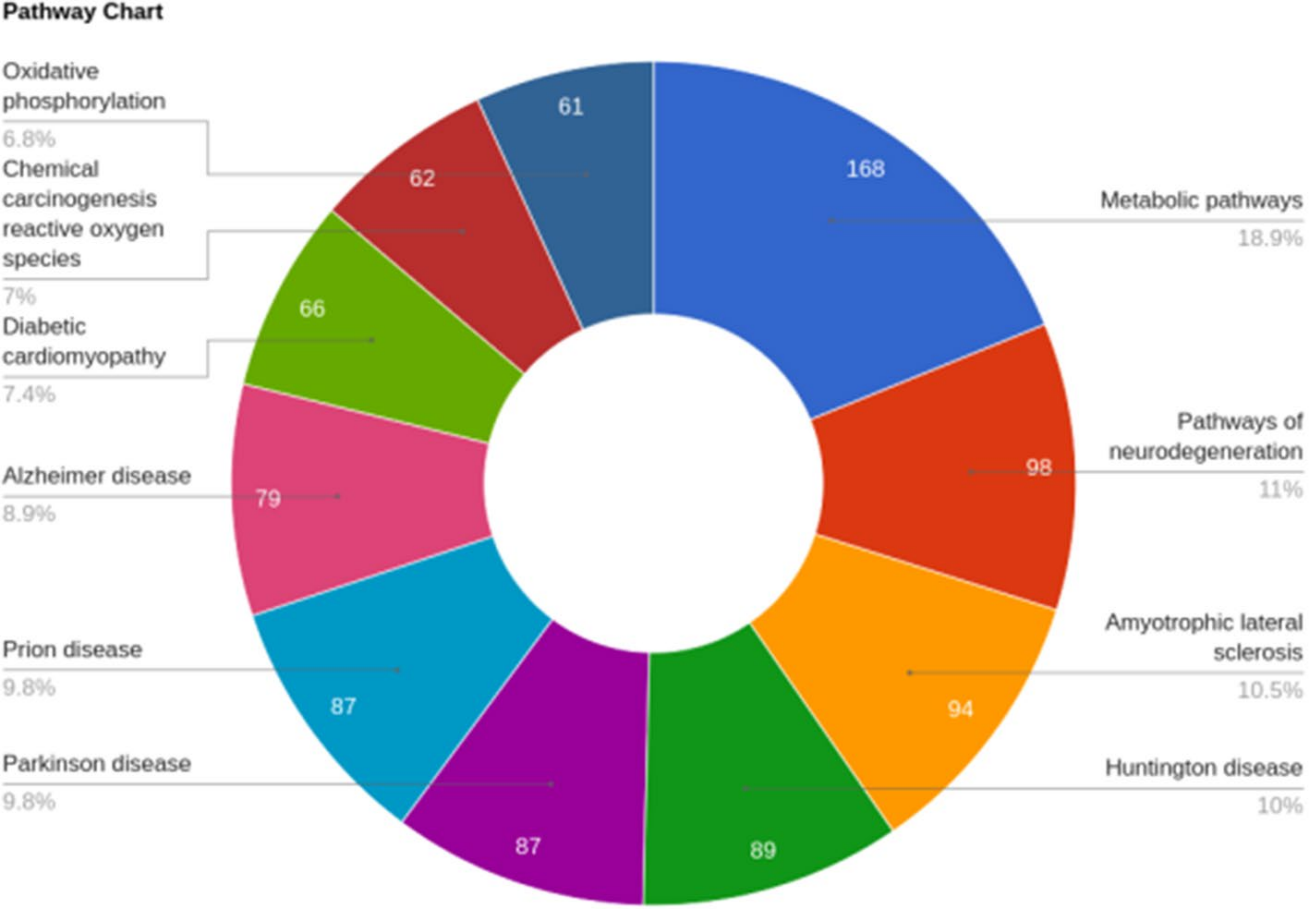
KEGG pathway analysis of global proteome of buffalo spermatozoa. Top 10 pathways wherein the identified proteins are involved are indicated in the figure. Permission has been obtained from Kanehisa laboratories for using KEGG pathway database^61^.

We identified a total of 1305 proteins in buffalo spermatozoa using LC–MS/MS approach. The proteomic profile of spermatozoa from individual bulls is depicted in Fig. 1 and the total list of proteins identified in buffalo spermatozoa are given in Supplementary File 1. Gene Ontology (GO) analysis of buffalo sperm proteins revealed their involvement in 122 biological processes (BPs) including several important sperm-fertility associated processes like glycolytic process, mitochondrial respiratory chain, tricarboxylic acid cycle, protein folding, spermatogenesis, sperm motility and sperm binding to zona pellucida. GO analysis recognized that the identified proteins were involved in 83 cellular components (CCs) with 26% of the proteins localized in cytoplasm, 14% in mitochondria and 6% in membrane. Among the 99 molecular functions (MFs) wherein the identified proteins were involved, 10% of the proteins were found to be involved in ATP binding while the other important function includes metal ion binding, DNA binding, protein binding and ATPase activity. The top 10 BPs, CCs and MFs in which the identified proteins are involved are given in Fig. 2. KEGG pathway enrichment revealed that the identified proteins were involved in 68 different pathways; the top 10 pathways are indicated in Fig. 3. The principle KEGG pathways wherein sperm proteins were found to be enriched include metabolic pathways (168 proteins), neurodegeneration (98 proteins), reactive oxygen species (62 proteins) and oxidative phosphorylation (61 proteins).

**Figure 4:**
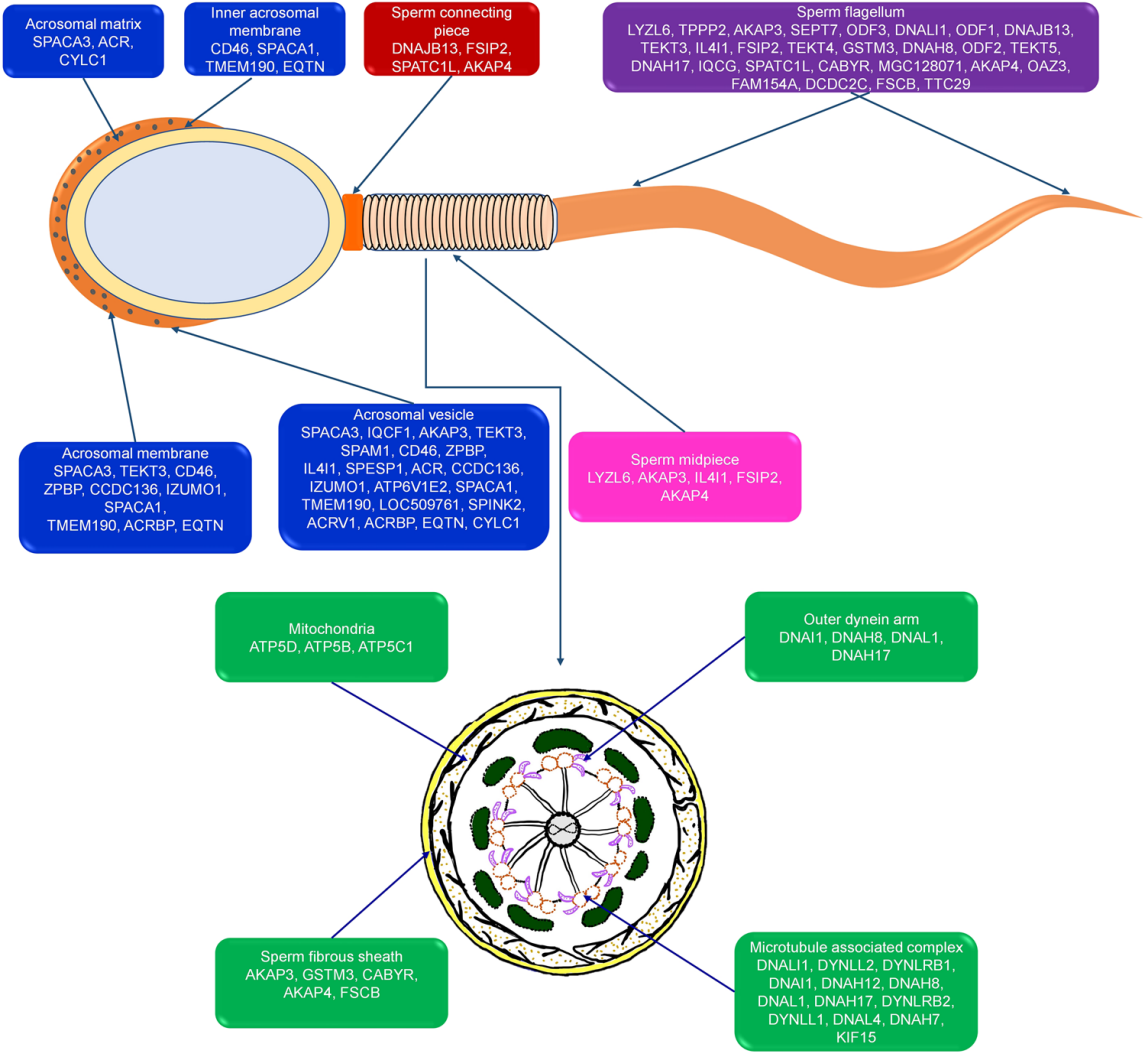
Subcellular localisation of the important proteins identified in buffalo bull spermatozoa.

The proteins significantly (P < 0.05) associated with spermatogenesis and different sperm functions are sorted out from the list of total proteins identified. It was found that several proteins were associated with spermatogenesis, sperm motility apparatus, capacitation, sperm-zona binding and fertilization (Table 1). The subcellular localization of proteins involved in regulating sperm functions is indicated in Fig. 4.

**Table 1:** Significant proteins detected in global proteomic analysis of buffalo spermatozoa and their association with important sperm functions and fertility.

Sperm functions	Proteins	p value
Spermatogenesis	DRC7, ACE, ODF1, ODF2, ODF3, SPATC1L, HSPA2, SMRP1, SEPTIN7, OAZ3, SOD1, TPPP2, AXL, SLC25A31, CYLC2, CYLC1, SPATA19	0.004
Spermatid development	PACRG, TSSK1B, CCDC63, CCDC42, SPINK2B, HSPA2, IQCG	0.020
Sperm axoneme assembly	DRC7, CFAP157, CFAP97D1, IQCG, FSIP2	0.006
Motile cilium assembly	RSPH6A, RSPH9, AKAP4, CCDC40	0.030
Cilium movement	RSPH6A, TEKT1, TEKT2, TEKT3, TEKT4, RSPH9, TEKT5	4.13E−06
Progressive regulation of sperm motility	TF, PHPT1, CFAP20	0.029
Flagellated sperm motility	DRC7, SORD, AKAP4, ENKUR, LDHC, TEKT2, TEKT3, TEKT4, ROPN1L, TEKT5, DNAI1, ROPN1, GAS8, CCDC40, RAC1, ACTB	5.87E−08
Sperm capacitation	CABYR, ROPN1L, ELSPBP1, PRKACA, DLD, ROPN1	0.001
Sperm egg recognition	IZUMO1, SPESP1, SPACA3	0.029
Sperm binding to zona pellucida	CCT3, CCT2, ARSA, HSPA1L, TCP1, GLIPR1L1, CCT7, SPA17, ALDOA, CCT5, ZPBP, CCT4	7.74E−08
Fusion of sperm to egg plasma membrane	IZUMO1, SPESP1, EQTN, SPACA3, LOC509761, SPAM1, LYZL6	1.56E−05

### Differential proteomic profile of spermatozoa from high- and low-fertile buffalo bulls

**Figure 5:**
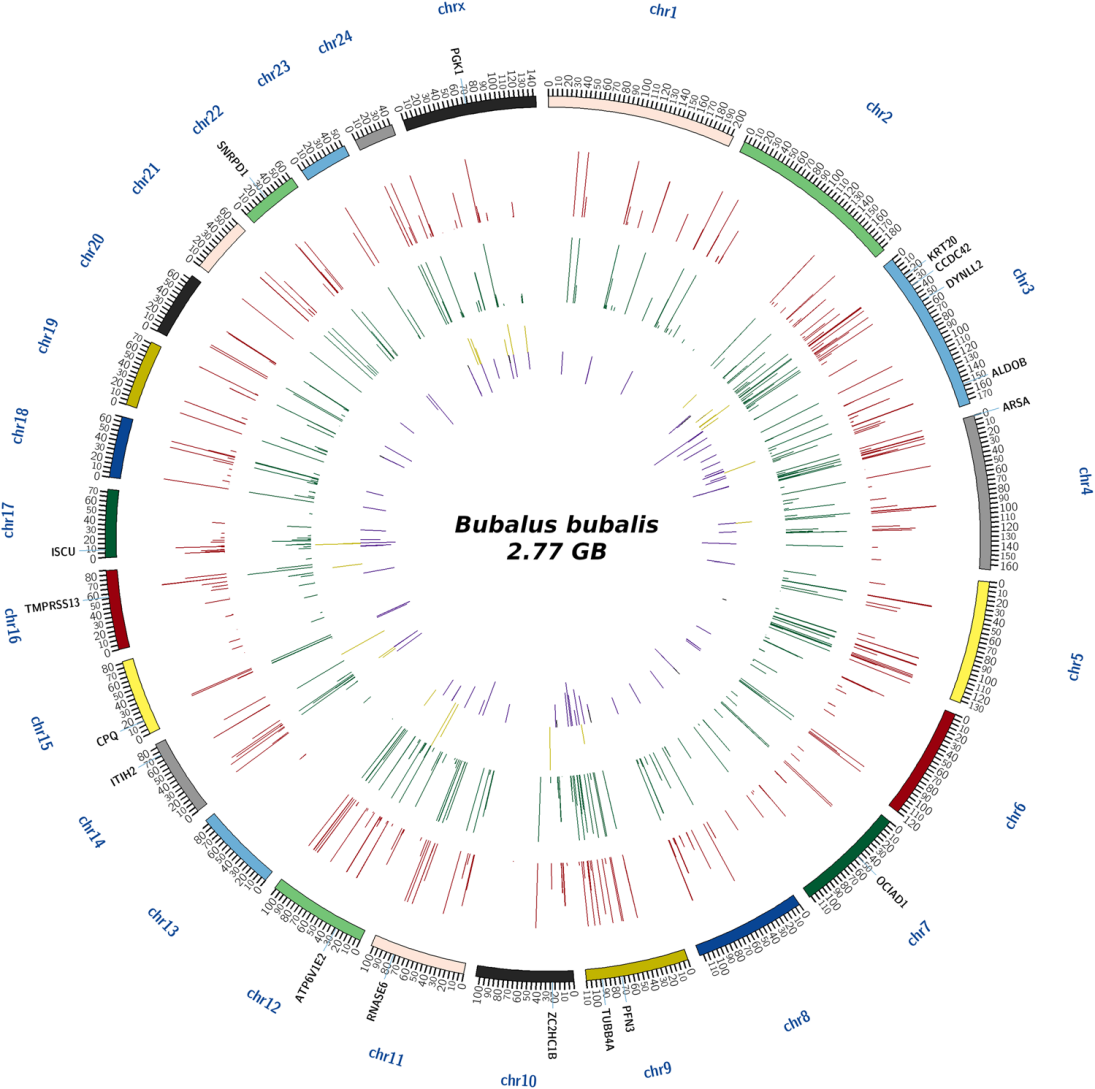
The Circos Plot represent the reference genome. The outer circle is chrmosomal region. Inside the chromosomal circle, 1st circle highlights the protein abundance in BL sample. 2nd circle in green represent abundance of protein in BH sample and inner most circle is differentially expressed proteins. Circos plot was generated using the standalone tool “Circos”v0.69-8 (http://circos.ca/).

Among the proteins identified in buffalo bull spermatozoa, 1127 and 1006 were expressed in high- and low-fertile bulls, respectively. After filtering the data with a cut off of PSM > 2 and removing the non-abundant proteins, we arrived at a total of 973 proteins for differential proteomic analysis. It was observed that 844 proteins were commonly expressed in spermatozoa from both the groups while 77 (7.91%) and 52 (5.34%) proteins were exclusively expressed in high- and low-fertile bulls, respectively (Supplementary Fig. 1). The differentially expressed proteins between the high- and low-fertile buffalo bulls were identified by calculating the log2 of abundance ratio (fold change). Among the 844 commonly expressed proteins, 75 were upregulated (> 1-fold change) and 176 were downregulated (< − 1-fold change) in low- compared to high-fertile buffalo bulls, while 593 proteins were neutrally expressed (between 1 and – 1-fold change) in both the groups. The global proteomic profile was mapped against the *Bubalus bubalis* genome for chromosomal coverage. The abundance of the identified proteins and their differential expression are shown in Circos plot (Fig. 5).

**Figure 6:**
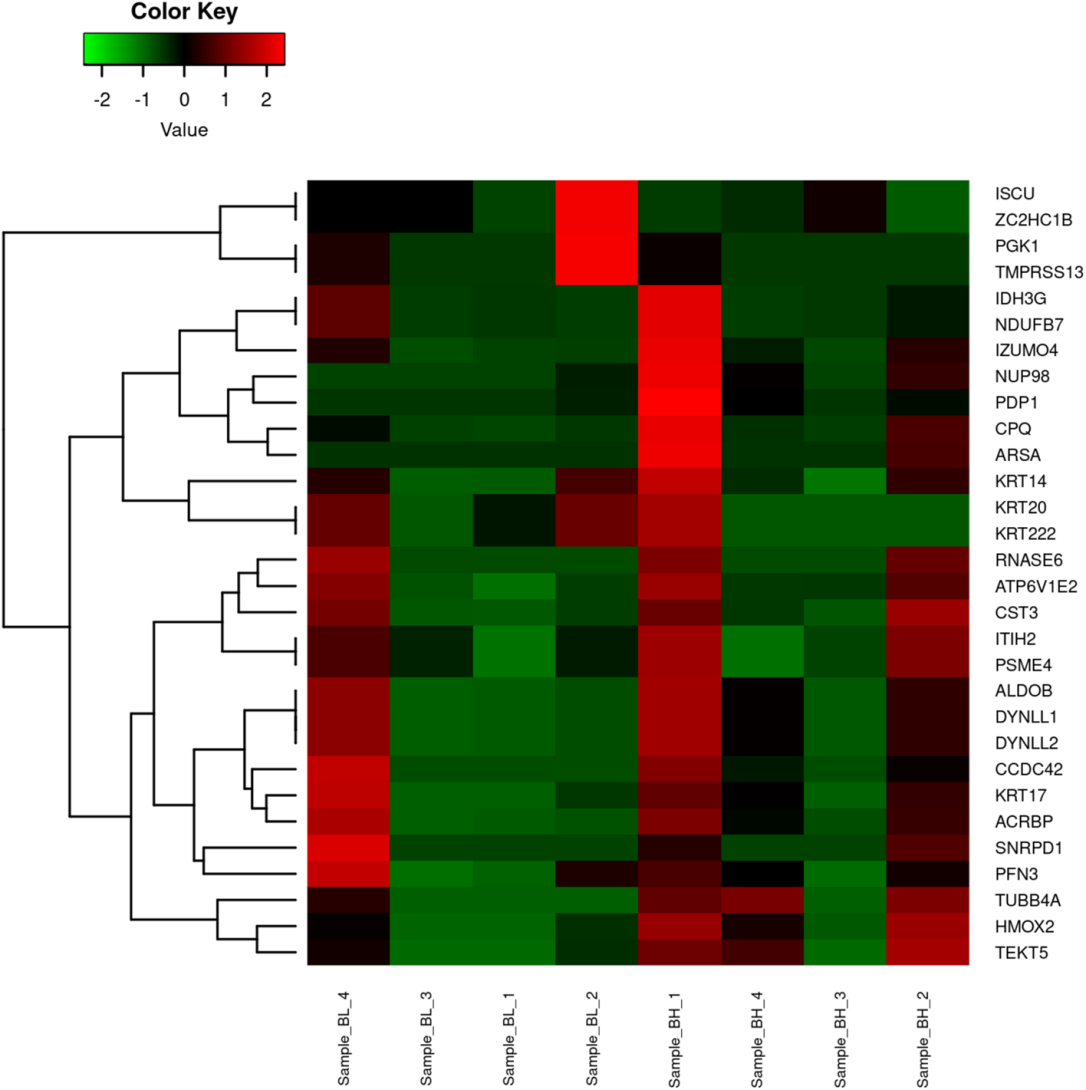
Heat map indicating the extent of differential expression of top 30 dysregulated proteins (15 upregulated and 15 downregulated) between high- and low-fertile buffalo bull spermatozoa. Heatmap was generated using R package “gplots” v3.1.3 (https://github.com/talgalili/gplots).

Top differentially expressed proteins (DEPs) with the highest upregulated expression in low-fertile bulls included Proteasome activator complex subunit 4, Inter-alpha-trypsin inhibitor heavy chain H2, Iron-sulfur cluster assembly enzyme, Zinc finger C2HC domain-containing protein 1B, Keratin, type I cytoskeletal proteins, V-type proton ATPase subunit E 2 and Fructose-bisphosphate aldolase B (Table 2). Among the DEPs, Coiled-coil domain-containing protein 42, Tubulin beta-4A chain, Small nuclear ribonucleoprotein, Arylsulfatase A, Cystatin-C, Ribonuclease K6, Profilin-3, Carboxypeptidase Q, Dynein light chain and NADH dehydrogenase 1 beta subcomplex subunit 7 were highly downregulated in low-fertile bulls (Table 3). The differential expression heatmap of top 15 upregulated and downregulated sperm proteins in low-fertile buffalo bulls is given in Fig. 6.

**Table 2:** List of 15 significantly up-regulated proteins in spermatozoa of low-fertile buffalo bulls.

Uniprot_ID	Gene name	Description	Log2 fold change
F1MKX4	PSME4	Proteasome activator complex subunit 4 (proteasome activator PA200)	3.32
A5D7R6	ITIH2	ITIH2 protein (inter-alpha-trypsin inhibitor heavy chain H2)	3.25
Q17QE6	ISCU	Iron-sulfur cluster assembly enzyme (iron-sulfur cluster scaffold homolog)	3
Q32KN7	ZC2HC1B	Zinc finger C2HC domain-containing protein 1B	2.86
A1L595	KRT17	Keratin, type I cytoskeletal 17	2.59
Q2KI75	KRT222	Keratin-like protein KRT222 (keratin-222)	2.47
F1MPK1	KRT20	Keratin, type I cytoskeletal 20 (cytokeratin-20) (CK-20) (keratin-20) (K20)	2.47
P05785	KRT14	Keratin, type I cytoskeletal 14	2.47
Q32LB7	ATP6V1E2	V-type proton ATPase subunit E 2 (V-ATPase subunit E 2) (vacuolar proton pump subunit E 2)	2.46
Q3T0S5	ALDOB	Fructose-bisphosphate aldolase B (EC 4.1.2.13) (liver-type aldolase)	2.18
Q9GK71	PGK1	Phosphoglycerate kinase 1 (EC 2.7.2.3)	2.05
V6F7U3	TMPRSS13	Transmembrane protease, serine 13-like (Transmembrane serine protease 13)	1.97
F1MGH5	TEKT5	Tektin-5	1.89
Q3T0J4	HMOX2	Heme oxygenase (biliverdin-producing) (EC 1.14.14.18)	1.8
Q58DM0	IDH3G	Isocitrate dehydrogenase [NAD] subunit, mitochondrial	1.78

**Table 3:** List of 15 significantly down-regulated proteins in spermatozoa of low-fertile buffalo bulls.

Uniprot ID	Gene name	Description	Log2 fold change
A6QPE2	IZUMO4	MGC148336 protein	− 2.08
E1BCV4	NUP98	Nuclear pore complex protein Nup98-Nup96	− 2.09
E1B7S8	ACRBP	Acrosin-binding protein (acrosin-binding protein, 60 kDa form) (proacrosin-binding protein sp32)	− 2.12
P35816	PDP1	[Pyruvate dehydrogenase [acetyl-transferring]]-phosphatase 1, mitochondrial (PDP 1) (EC 3.1.3.43) (protein phosphatase 2C) (pyruvate dehydrogenase phosphatase catalytic subunit 1) (PDPC 1)	− 2.16
Q02368	NDUFB7	NADH dehydrogenase [ubiquinone] 1 beta subcomplex subunit 7 (Complex I-B18) (CI-B18) (NADH-ubiquinone oxidoreductase B18 subunit)	− 2.26
Q3MHR3	DYNLL2	Dynein light chain 2, cytoplasmic	− 2.32
Q862K7	DYNLL1	Dynein light chain (fragment)	− 2.32
Q17QK3	CPQ	Carboxypeptidase Q (EC 3.4.17.-) (plasma glutamate carboxypeptidase)	− 2.39
Q32PB1	PFN3	Profilin-3	− 2.4
P08904	RNASE6	Ribonuclease K6 (RNase K6) (EC 3.1.27.-) (K6b) (ribonuclease K2) (RNase K2)	− 2.42
P01035	CST3	Cystatin-C (colostrum thiol proteinase inhibitor) (cystatin-3)	− 2.43
Q08DD1	ARSA	Arylsulfatase A (ASA) (EC 3.1.6.8) (cerebroside-sulfatase)	− 2.47
Q3ZC10	SNRPD1	Small nuclear ribonucleoprotein Sm D1 (Sm-D1) (snRNP core protein D1)	− 2.59
Q3ZBU7	TUBB4A	Tubulin beta-4A chain (tubulin beta-4 chain)	− 2.92
A6QQM8	CCDC42	Coiled-coil domain-containing protein 42	− 3.9

### Gene set enrichment and pathway analysis of DEPs

**Figure 7:**
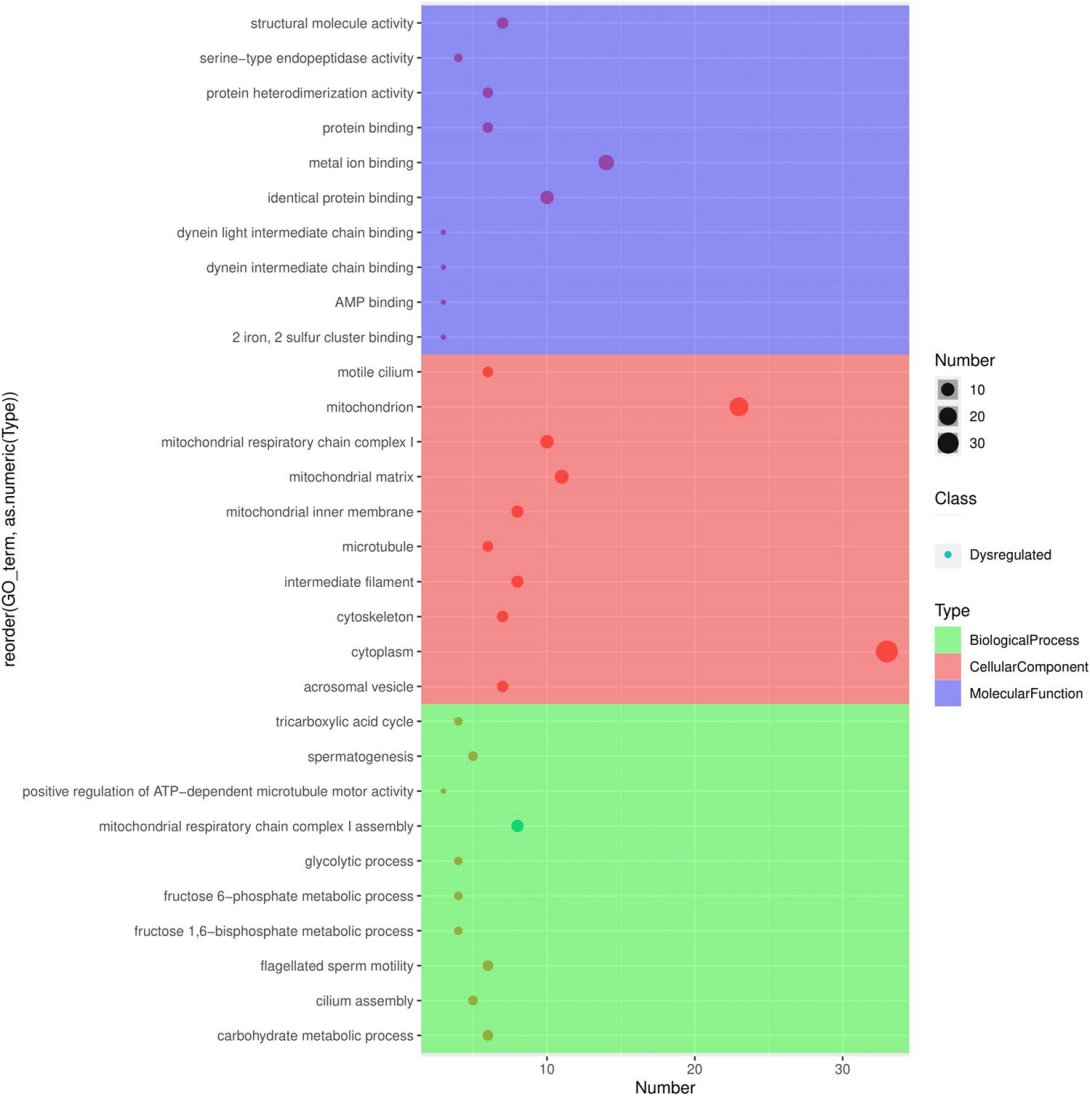
Gene Ontology of differentially expressed proteins between high- and low-fertile of buffalo bull spermatozoa. Top 10 GO terms (biological process, cellular component and molecular function) are indicated in the figure. Gene Ontology bubble plot was generated using "ggplot2" version 3.3.6 (https://ggplot2.tidyverse.org/).

**Figure 8:**
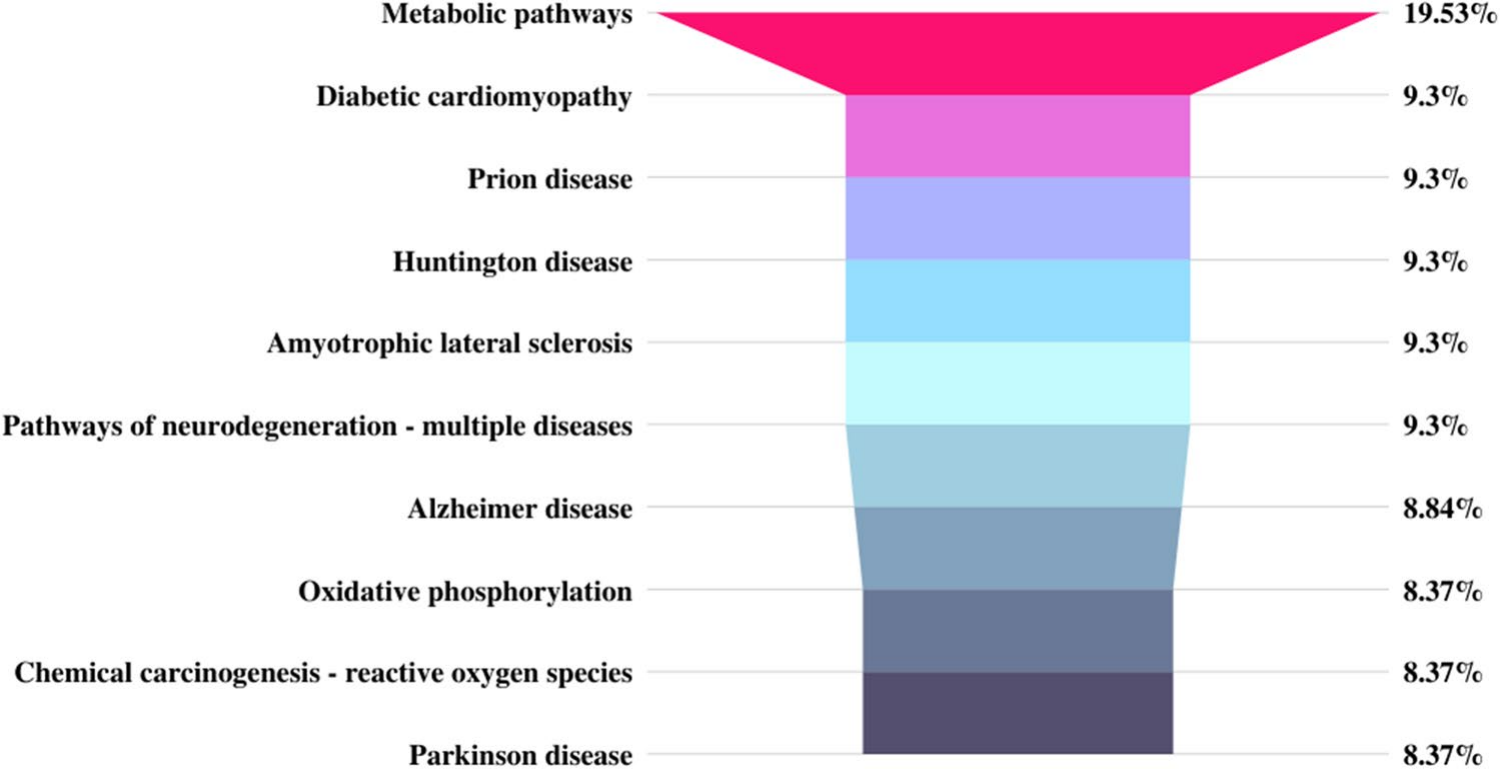
KEGG pathway analysis of differentially expressed proteins between high- and low-fertile of buffalo bull spermatozoa. Top 10 pathways wherein the differentially expressed proteins are involved are indicated in the figure. Permission has been obtained from Kanehisa laboratories for using KEGG pathway database^61^.

Bioinformatics functional analysis was performed over the differentially expressed proteins. Overall, 76 GO terms (33 biological processes, 19 molecular functions, and 24 cellular components) and 38 KEGG pathways were enriched among the sperm proteins differentially expressed between high- and low-fertile buffalo bulls. Among the enriched biological processes were mitochondrial respiratory chain complex I assembly, flagellated sperm motility, carbohydrate metabolic process, cilium assembly and spermatogenesis. Among the molecular functions, the prominent ones included metal ion binding, identical protein binding and structural molecular activity. The top 10 BPs, CCs and MFs in which the DEPs were involved are given in Fig. 7. Pathway enrichment of differentially expressed sperm proteins indicated that the DEPs were found to be enriched in 39 pathways including metabolic pathways (42 proteins), oxidative phosphorylation (18 proteins) and reactive oxygen species (18 proteins). The top 10 enriched pathways are shown in Fig. 8.

### Functional analysis of upregulated proteins in low-fertile bulls

**Figure 9:**
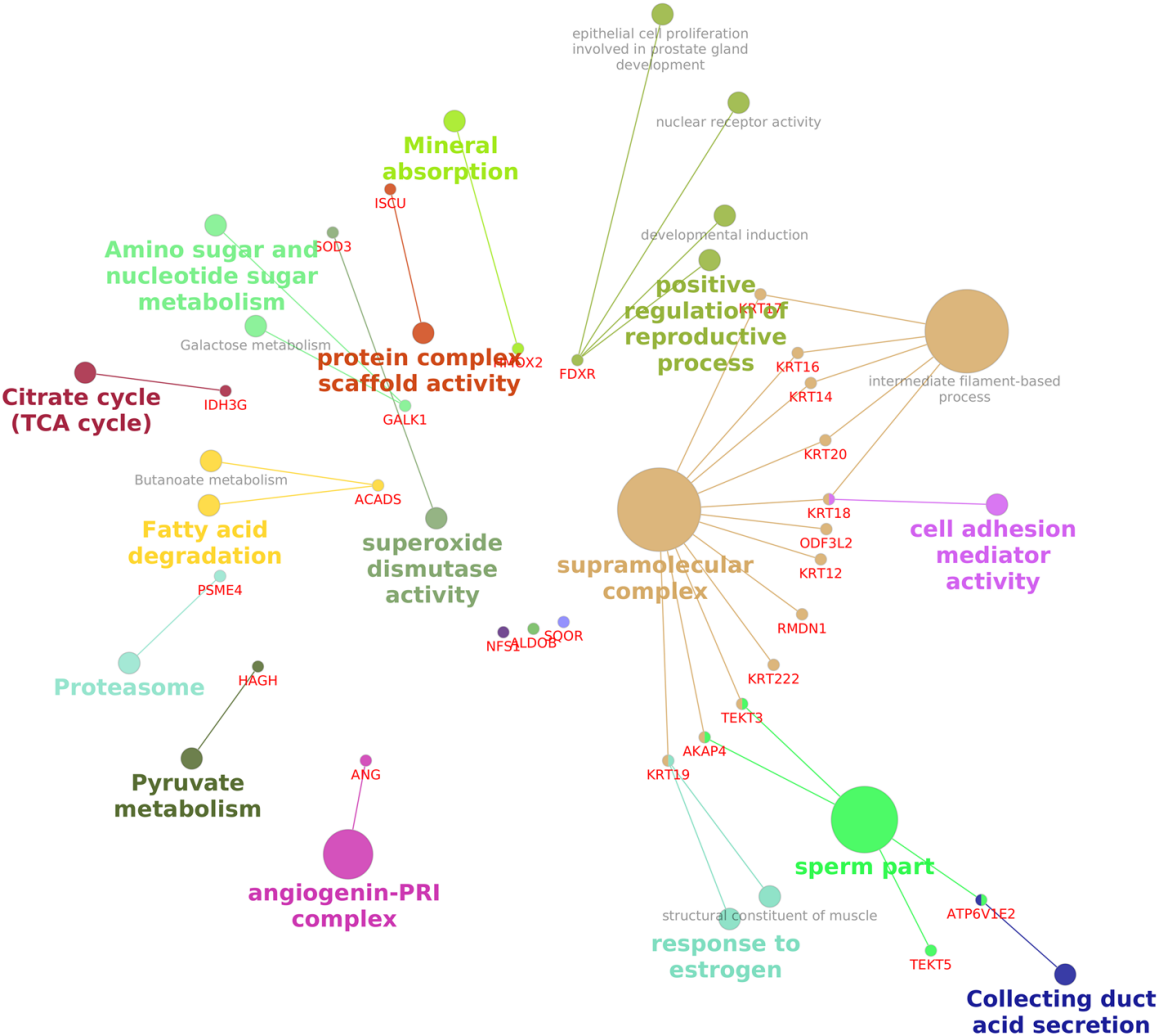
Protein–protein interaction network of upregulated proteins in low-fertile buffalo bull spermatozoa. Interaction network was generated using “Cluego” v2.5.8 package (https://apps.cytoscape.org/apps/cluego).

Functional annotation of upregulated proteins in the spermatozoa from low-fertile bulls revealed that they were involved in 11 BPs, 7 CCs and 5 MFs. KEGG pathway enrichment of the upregulated proteins of high-fertile group revealed that these proteins were involved in 7 different pathways. The list of significantly upregulated proteins in low-fertile bulls is given in Supplementary File 2. The upregulated proteins in low-fertile group were involved in various BPs like chromatin silencing, flagellated sperm motility, response to hypoxia and cilium movement in cell motility. In addition, upregulated proteins are involved in MFs like protein heterodimerization activity, DNA binding, structural molecular activity and enzyme binding. Upon Network bioinformatics analysis, it was identified that upregulated proteins were involved in regulation of reproductive process, supramolecular complex, sperm part and response to oestrogen (Fig. 9).

### Functional analysis of downregulated proteins in low-fertile bulls

**Figure 10:**
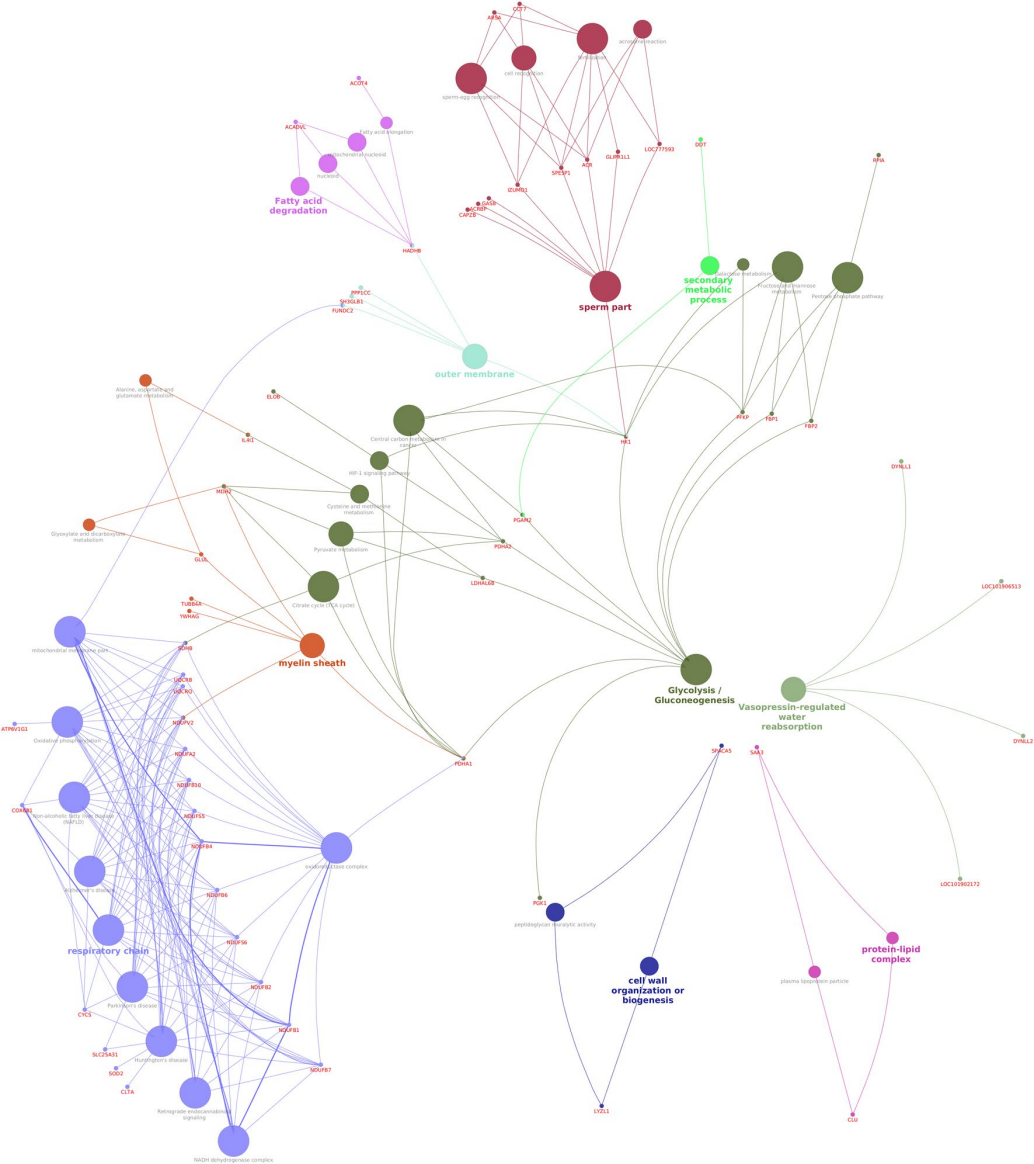
Protein–protein interaction network of downregulated proteins in low-fertile buffalo bull spermatozoa. Interaction network was generated using “Cluego” v2.5.8 package (https://apps.cytoscape.org/apps/cluego).

Functional annotation of downregulated proteins in the spermatozoa from low-fertile bulls revealed that they were involved in 28 BPs, 20 CCs and 13 MFs. KEGG pathway enrichment of the downregulated proteins of low-fertile group revealed that these were involved in 31 different pathways. The list of significantly upregulated proteins in low-fertile bulls is given in Supplementary File 3. The down regulated proteins in low-fertile group were found to be enriched in various BPs like mitochondrial respiratory chain complex, microtubule-based process, positive regulation of ATP-dependent microtubule motor activity, fructose 6-phosphate metabolic process, fertilization process, binding of sperm to zona pellucida, flagellated sperm motility, sperm-egg recognition, acrosome reaction and fusion of sperm to egg plasma membrane. In addition, the downregulated proteins were found to be involved in MFs like protein binding, protein heterodimerization activity, dynein light intermediate chain binding, fructose 1,6-bisphosphate 1-phosphatase activity and pyruvate dehydrogenase (acetyl-transferring) activity. Pathway enrichment analysis showed that these proteins are involved in various important pathways like Metabolic pathways, oxidative phosphorylation, reactive oxygen species, retrograde endocannabinoid signalling and fructose metabolism. Upon network bioinformatics analysis, it was identified that downregulated proteins of low-fertile group were involved in glycolysis/gluconeogenesis, oxidoreductase complex, myelin sheath, sperm part, fatty acid degradation and secondary metabolic process (Fig. 10).

### Functional network analysis of dysregulated proteins

**Figure 11:**
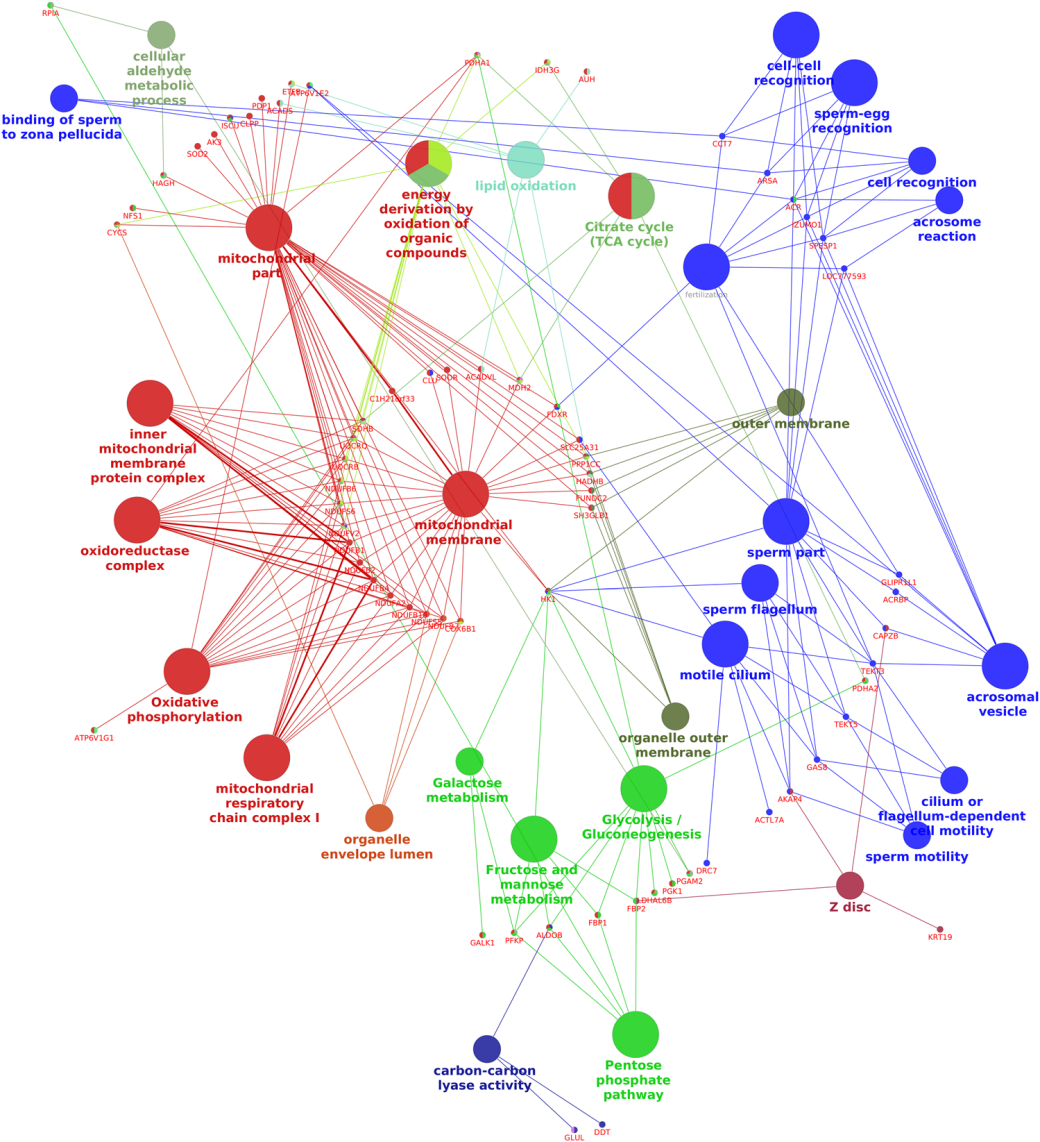
Cytoscape functional interaction network of differentially expressed proteins between high- and low-fertile of buffalo bull spermatozoa. Interaction network was generated using “Cluego” v2.5.8 package (https://apps.cytoscape.org/apps/cluego).

Protein–protein interaction network created using STRING interaction software revealed the cluster genes interacting with each other (Supplementary Fig. 2). Network analysis of the total dysregulated proteins in low-fertile buffalo bulls was carried out to find out the functional interactions of GO and pathways in which those proteins were involved. It was interesting to note that several dysregulated proteins in low-fertile bulls were found to interact among themselves and involved in several processes that are important for sperm functions and fertility including mitochondrial membrane potential, oxidative phosphorylation, sperm flagellar activity, acrosome reaction and sperm-zona pellucida binding (Fig. 11).

## Discussion

Buffaloes play an important role in global milk and meat production, and livelihood and nutritional security of a significant proportion of human population depends upon this species; yet their full production and reproduction potential has not been achieved. One of the major reasons, often cited, for reduced reproductive performance in buffaloes is poor conception rates with artificial insemination. Like in case of cattle, several thousands of buffaloes are artificially inseminated using semen from a single bull; therefore, bull fertility is an important determinant of successful reproduction. On the other hand, buffalo bull fertility has not been understood properly; only very few studies are available on sperm molecular health and bull fertility in this species^7^. In the present study, we carried out an in-depth proteome profiling of buffalo bull spermatozoa and identified potential fertility associated sperm proteins that could serve as marker for selection of high-fertile buffalo bulls for artificial breeding.

### Global proteomic profile of buffalo spermatozoa

Using LC–MS/MS approach, we identified a total of 1305 proteins in buffalo spermatozoa. A majority of the identified proteins were found to be enriched in important sperm fertility associated processes like spermatogenesis, metabolism, sperm motility, capacitation, acrosome reaction and sperm binding to zona pellucida, among others.

We identified that several proteins related to spermatogenesis and sperm development were found in higher abundance in buffalo spermatozoa. Testis specific Angiotensin Converting Enzyme (ACE) is important for fertility; sperm from mice lacking ACE isozymes show defects in transport within the oviducts and in binding to zonae pellucida^22^. Proper distribution of ACE in the neck and mid-piece is required for normal sperm motility that could be used as a novel biomarker for male infertility^23^. We identified that Outer dense fiber proteins (ODF1, ODF2 and ODF3) were found in high abundance in buffalo spermatozoa. ODFs are the major protein of the mammalian sperm tail outer dense fibers. Impaired development of the ODFs is a major cause of tail abnormalities in infertile men, indicating an important function in sperm motility and/or morphology^24,25^. We found that testis-specific HSPA2 (Heat Shock Protein Family A (Hsp70) Member 2) was in abundance in buffalo spermatozoa. It has been reported that reduced expression of HSPA2 in humans is associated with oligozoospermia^26^, abnormal sperm morphology^27^, aberrant histone-protamine replacement and low zona pellucida binding ability^28^, all of which can result in reduced fertility. Ornithine decarboxylase antizyme 3 (OAZ3), observed in high abundance in buffalo spermatozoa, is a testis-specific antizyme paralog and the only antizyme expressed in the mid to late stages of spermatogenesis. OAZ3 is the only antizyme present in post-meiotic male germ cells, which might play an important role in maintaining spermatogenesis by controlling ODC and polyamine levels^29^. We found that the expression of SPATC1L (Spermatogenesis and Centriole Associated 1 Like) protein was high in buffalo spermatozoa. Earlier, it was reported that the expression of SPATC1L start from spermatids and the protein is localized to the neck region in testicular sperm. This protein plays an important role in spermatogenesis and male mice lacking SPATC1L were completely sterile owing to separation of sperm heads from sperm tails^30^. The coiled-coil domain-containing (CCDC) proteins have been implicated in a variety of physiological and pathological processes including sperm motility, functions and fertility. CCDC42 is expressed in spermatids and localizes to the manchette, the connecting piece and the tail. It was hypothesised that the protein complex consisting of the core proteins ODF2/ODF1/CCDC42 may build the rigid scaffold essential for the formation of the connecting piece and the sperm tail. When any one of these proteins is missed, the rigid scaffold is damaged causing failure of the linkage complex and the sperm tail^31^.

Impaired sperm motility is considered as one of the primary causes of male infertility^32^, and therefore it is essential to know the proteins associated with sperm motility. Many cilia, flagella-associated proteins and dynein family proteins have been reported to play a functional role in sperm motility^33^. In buffalo spermatozoa, we identified such proteins in abundance. Dynein Regulatory Complex Subunit 7 (DRC7) is a component of Nexin-Dynein Regulatory Complex, which is localized between doublet microtubules in sperm tail. It has been shown that sperm axoneme was disorganized in DRC7 knocked out mice and were infertile due to their short immotile spermatozoa^34^. Radial Spoke Head 6 Homolog A (RSPH6A) is an evolutionarily conserved testis-enriched protein localized in sperm flagellum and without this protein, sperm flagellar elongation may become unstable^35^. Recently, the importance the protein A-Kinase Anchoring Proteins gained momentum because of its association with bull fertility. We found that AKAP4 was abundantly present in buffalo spermatozoa. AKAP4 protein is the most abundant constitutive protein of the sperm fibrous sheath in all mammals and is phosphorylated by tyrosine kinase which triggers a cascade of protein phosphorylation involved in sperm motility and hypermotility^36^. Tetkins (TEKT) are constitutive proteins of microtubules in cilia; five types of mammalian TEKTs have been reported, all of which have been verified to be present in sperm flagella. Among the five types, TEKT2 is indispensable for sperm structure, mobility, and fertilization^37^. In the present study, we identified that all the five types of Tetkins are abundantly present in buffalo spermatozoa.

In buffalo spermatozoa, we identified that the proteins (CABYR, ROPN1L, ELSPBP1, PRKACA, DLD, ROPN1) associated with sperm capacitation were expressed abundantly. Calcium-binding tyrosine phosphorylation-regulated protein (CABYR) localizes to the principal piece of the sperm flagellum and is the first demonstration of a sperm protein that gains calcium-binding capacity when phosphorylated during capacitation^38^. Rhophilin Associated Tail Proteins (ROPN) are involved in sperm capacitation. It has been reported that ROPN1 resides in sperm flagella and is functionally involved in motility regulation, therefore increased ROPN1 abundance might help the movement of capacitated spermatozoa towards the ovum for fertilization^39^. We identified that ROPN1 proteins were abundantly expressed in buffalo spermatozoa. We observed that several proteins involved in sperm-zona binding and fusion of sperm to egg plasma membrane were abundantly detected in buffalo spermatozoa. Izumo sperm-egg fusion 1 (IZUMO1) is a membrane protein that localizes to the acrosomal membrane and is required for binding of the acrosome-reacted spermatozoa to oolemma prior to fusion. IZUMO1 knocked out spermatozoa can penetrate the zona pellucida but cannot fuse with the egg plasma membrane, resulting in male sterility^40^. Sperm equatorial segment protein 1 (SPESP1) is concentrated in the equatorial segment of mature sperm. It has been shown that SPESP1 is involved in sperm-egg fusion and sperm from mice bearing a targeted disruption of the SPESP1 gene showed a reduced ability to fuse with oocytes^41^. In buffalo spermatozoa, we observed that Chaperonin-containing tailless complex proteins (CCT2, CCT3, CCT4, CCT5 and CCT7) were expressed in abundance. These subunits of the TRiC complex are involved in the binding of capacitated spermatozoa to the zona pellucida^42^. GLIPR1L1 protein, which is found to be in high abundance in buffalo spermatozoa, is required for optimal fertilization at the stage of sperm-oocyte fusion and also has a role in optimizing acrosome function, the translocation of IZUMO1 during the acrosome reaction, and the fertilization process^43^. Zona pellucida binding protein (ZPBP), which are important acrosomal matrix components involved in interacting with the ZP during sperm penetration were also found to be abundantly present in buffalo spermatozoa.

We found that most of the sperm proteins identified in this study in buffalo spermatozoa were found to be relevant in the context of male fertility. Therefore, we compared the sperm proteome of high-fertile buffalo bulls with the sperm proteome of low-fertile buffalo bulls with the aim to identify fertility associated proteins, which are detailed below.

### Comparative proteomic analysis of high- and low-fertile buffalo bull spermatozoa

After filtering the data based on PSM and after removing the non-abundant proteins, we identified that 844 sperm proteins were common to both high- and low-fertile bulls, while 77 and 52 proteins were specific to high- and low-fertile buffalo bulls, respectively. Among the 844 commonly expressed proteins, 75 were upregulated and 176 were downregulated in low-compared to high-fertile buffalo bulls. We observed that most of the dysregulated proteins in low-fertile bulls were involved in metabolic pathways, oxidative phosphorylation and reactive oxygen species pathways. Network analysis also indicated that the dysregulated proteins were involved in several important sperm functions and fertility including mitochondrial membrane potential, oxidative phosphorylation, sperm flagellar activity, acrosome reaction and sperm-zona pellucida binding.

Sperm motility is a prime requirement for the spermatozoa to reach the site of fertilization and for fertilization of oocyte. We identified that sperm expression of NUP98, PDP1, DYNLL1, DYNLL2, CPQ, PFN3, CST3 and SNRPD1 (all are associated with sperm motility) were significantly downregulated in low-fertile buffalo bulls. The protein NUP98 (Nucleoporin 98 Precursor) is reported to regulate bipolar spindle assembly in spermatozoa through association with microtubules and its abundance was higher in high-motile than low-motile spermatozoa^44^. Yang et al.^45^ reported that decreased expression of PDP1 (Pyruvate Dehydrogenase Phosphatase Catalytic Subunit 1) may lead to impairment in ATP synthesis and inhibit AMPK activation. Dynein Light Chain proteins (DYNLL) is localized in the outer dynein arm, a molecular complex that drives the beating motion of cilia/flagella (Tanner et al., 2008). Carboxypeptidase Q (CPQ) is located mainly in the sperm midpiece and tail, may play an important role in the hydrolysis of circulating peptide, thereby suggesting its possible role in sperm motility^46^. Profilins (PFNs) are key regulatory proteins for the actin polymerization in cells and it has been reported that PFN knocked out sperm display abnormal manchette development leading to an amorphous sperm head shape. Additionally, the spermatozoa had reduced motility resulting from flagellum deformities^47^. Among the upregulated proteins in low-fertile buffalo bulls, the expression of PSME4 (Proteasome activator complex subunit 4) was significantly high (3.2 folds). It has been reported that PSME4 knockout male mice, although showed spermatogenesis defects, but were fertile. On the other hand, PSME3/PSME4 double knockout mice had significantly reduced sperm motility and were completely infertile^48^ indicating that higher expression of PSME4 alone might not significantly alter the sperm motility. The expression of Inter-alpha-trypsin inhibitor heavy chain H2 (ITIH2) was also highly upregulated (3.25 folds) in low-fertile buffalo bulls, however the function of this protein on sperm functions have not been reported. Another protein, ICSU (iron-sulfur cluster assembly protein) was significantly upregulated in low fertile bull spermatozoa. These proteins were reported to play a role in longevity and stress response in somatic cells^49^, however the role of these proteins in sperm functions is yet to be elucidated.

Appropriate expression of sperm proteins involved in sperm structure, acrosome reaction and zona binding are important for male fertility. In the current study, we observed that the expression of sperm proteins IZUMO4, ACRBP, ARSA and RNASE3, which are involved in acrosome reaction, fertilization and post-fertilization events were significantly downregulated in low-fertile buffalo bulls. Izumo sperm-egg fusion protein 4 (IZUMO4) is a soluble protein expressed in the testis and is a part of a multiprotein family whose members form large complexes on mammalian sperm and involved in sperm-egg fusion^50^. Acrosin binding protein (ACRBP) are largely necessary to induce capacitation, the acrosome reaction and sperm-zona pellucida binding, all of which are necessary steps for fertilization because sperm binding with zona pellucida is inhibited by anti-ACRBP antibodies^51^. Ribonuclease A Family Member 3 (RNASE3) has a role in post-fertilization development and it has been reported that the loss of paternal RNase 3 plays a role in miscarriage^52^. We observed that the protein expression of several keratins, including KRT14, 17, 20 and 222 were upregulated in low-fertile buffalo bull spermatozoa (2.47 folds). Earlier study also reported the presence of several keratins in Rhesus Macaque sperm, human sperm, rat sperm and mouse sperm^53,54^ suggesting that these proteins play important functional roles in sperm. Because the keratins not only serve structural roles, but might play a role in protein transport, restructuring, differentiation and proliferation^55^, any alterations in their expression could result in an abnormal sperm morphology and functions, which might affect the sperm fertility. However, the precise role of these keratins in bull sperm functions and fertility need to be elucidated.

Among the downregulated proteins in low-fertile buffalo bulls, ACRBP, CPQ, RNASE6, CST3 and ARSA were the secretory signal peptides transported by the Sec translocon and cleaved by Signal Peptidase I. Sec translocon represents an evolutionary conserved mechanism for delivering cytosolically-synthesized proteins to extra-cytosolic compartments, thereby indicates that these proteins could play vital roles in sperm functions. For instance, CST3 (Cystatin C or cystatin 3) is abundant in the testes, and has important implications for male reproduction. Reduced expression of CST3 decreases sperm viability and inhibits the signal initiating sperm capacitation (efflux of cholesterol from the sperm plasma membrane and protein tyrosine phosphorylation). Further, CST3 is also expressed in female reproductive tract to suppress sperm capacitation. Deficiency of CST3 results in decreased sperm motility via its effects on the mitochondrial metabolic pathways through Mitochondrial complex 1. Similarly, colocalization of Arylsulfatase A (ARSA) with the proteins of the complex (HSPA2/SPAM1) in the peri-acrosomal region is proposed as the mediator of sperm–zona pellucida binding^56^. Collectively, we observed that the expression of CST3, other proteins involved in mitochondrial respiratory chain complex I assembly and sperm capacitation (and acrosome reaction) associated proteins were significantly downregulated in low-fertile buffalo bulls, suggesting the alterations in sperm motility apparatus, sperm capacitation and associated events could be a reason for low fertility in these bulls.

In conclusion, we found that most of the sperm proteins identified in this study in buffalo bulls were found to be relevant in the context of male fertility. The sperm proteome data of buffalo bulls, generated in the study, will accelerate further research in the context of male fertility. The down regulated proteins in low-fertile group were found to be involved in important sperm functions required for fertility including mitochondrial respiratory chain complex, positive regulation of ATP-dependent microtubule motor activity, flagellated sperm motility, binding of sperm to zona pellucida, sperm-egg recognition, acrosome reaction and fusion of sperm to egg plasma membrane. Therefore, we believe that the differences in sperm proteome identified in the current study provides a platform to develop molecular tools to select buffalo bulls for fertility.

## Materials and methods

### Ethical statement

The present study was conducted at the Theriogenology Laboratory, Southern Regional Starion of ICAR-National Dairy Research Institute, Bengaluru, Karnataka India. All the experiments were conducted in accordance with the guidelines and regulations laid down and duly approved by Institute Animal Ethics Committee (CPCSEA/IAEC/LA/SRS-ICAR-NDRI-2019/No.04).

### Experimental bulls and fertility classification

Murrah buffalo bulls (n = 21) that qualified the breeding soundness evaluation and routinely used for artificial breeding were utilized for the study. The age of bulls ranged from 4 to 7 years and were maintained under uniform management conditions at the Institute’s farm. Vaccination, de-worming, regular check-up for communicable diseases and other herd-health programmes were followed as per the farm schedule and the bulls were free from major infectious diseases. Bull fertility was assessed based on the conception rates. Conception rates were calculated based on the number of buffaloes became pregnant out of the total number of buffaloes bred with the semen from an individual bull. The number of females inseminated using semen from the experimental bulls ranged from 102 to 196 per bull. All the female buffaloes were maintained under common management conditions and inseminations were carried out by an experienced veterinarian. The calculated conception rate was adjusted for non-genetic parameters including season, parity, stage of lactation and age of buffalo using the model given by Singh et al.^6^. The adjusted conception rate was used for the calculation of bull fertility. The bulls having conception rate more than mean + 1 standard deviation were considered as high-fertile (n = 4) and those having conception rate less than mean − 1 standard deviation were considered as low-fertile (n = 4). The mean conception rate of high- and low-fertile bulls was 43.4% and 19.2%, respectively and the difference was significant (p < 0.01).

### Sperm preparation and protein extraction

Spermatozoa from all the eight (4 high-fertile and 4 low-fertile) buffalo bulls were individually subjected to global proteomic analysis. Percoll gradient was used to obtain purified spermatozoa for proteomic analysis. For each bull, cryopreserved semen straws (produced from three different ejaculates and freezing operations) were thawed at 37 °C for 30 s and pooled. Purification of spermatozoa was done using 90–45% discontinuous Percoll gradient prepared in sperm TALP as per the procedure described in Saraf et al.^57^. Briefly, 0.9 mL of 45% of Percoll fraction was layered over 0.2 mL of 90% Percoll fraction for preparing gradient. Frozen thawed straw content was carefully layered over the top of prepared Percoll gradient fraction and subjected to centrifugation (950 g for 15 min). The resulting sperm pellet was then washed thrice using Phosphate Buffered Saline (PBS) before protein extraction.

Spermatozoa were lysed in 6 M Gn-HCl (50 mM TRIS, pH 8.8) buffer. Sperm proteins (50 µg) was used for digestion and reduced with 5 mM TCEP tris (2-carboxyethyl) phosphine), further alkylated with 50 mM iodoacetamide and then digested with Trypsin (1:50, Trypsin/lysate ratio) for 16 h at 37 °C. Digests were cleaned using a C18 silica cartridge to remove the salt and dried using a speed vac. The dried pellet was resuspended in buffer containing acetonitrile (2%) and formic acid (0.1%).

### Mass spectrometric analysis of peptide mixtures

Proteomic analysis experiment was performed using an EASY-nLC 1200 system (Thermo Fisher Scientific, USA) coupled to Thermo Fisher-QExactive equipped with nano electrospray ion source. Sperm proteins (1 µg) was loaded on C18 column of 50 cm, 3.0 μm Easy-spray column (Thermo Fisher Scientific™). Peptides were eluted using a 0–40% gradient of buffer consisting of 80% acetonitrile, 0.1% formic acid at a flow rate of 350 nl/min and injected for MS analysis. The obtained LC gradients were run for 100 minutes^58,59^. MS1 spectra were acquired in the Orbitrap at a resolution of 70 k. Dynamic exclusion was employed for 10 s excluding all charge states for a given precursor. MS2 spectra were acquired at rate of 17,500 resolutions^60^. All the samples were processed and RAW files generated were analyzed with Proteome Discoverer (v2.4) against the Uniprot Bovine reference proteome database. For Sequest and Amanda search, the precursor and fragment mass tolerances were set at 10 ppm and 0.5 Da, respectively. The protease used to generate peptides, i.e. enzyme specificity was set for trypsin/P (cleavage at the C terminus of K/R: unless followed by “P”) along with maximum missed cleavages value of two. Carbamidomethyl on cysteine as fixed modification and oxidation of methionine and N-terminal acetylation were considered as variable modifications for database search. Both peptide spectrum match and protein false discovery rate were set to 0.01 FDR.

### Data analysis

Raw abundance values were filtered on the basis of valid values (should be quantified in at-least 2/3 samples in each condition). Filtered values were log 2 standardized followed by imputation of missing values on the basis of normal distribution. The values were normalized followed by t-test. Significance was calculated using p-values. For differential statistical analysis, the abundance values of each sample were employed. Valid values were used to filter protein abundance values. Benjamini Hochberg FDR (cut-off 0.05) is used to calculate significance. For analysis, spermatozoa from high-fertile buffalo bulls were kept as control while spermatozoa from low-fertile buffalo bulls were considered as treatment. The fold change of expression levels (log base2) in comparison to control samples was used to identify differentially expressed proteins. Upregulated (> 1-fold) and downregulated (1-fold) proteins were considered as differentially expressed proteins. Significant DEP’s proteins were obtained by filtering based on the peptide spectrum matches (PSM’s) (PSM > 2) and removing the non-abundant protein (not found in both the groups). Multiple bioinformatics tools (DAVID, STRING and Cluego) were used to annotate DEPs using information from KEGG and Uniprot database for *Bubalus bubalis* and *Bos taurus* species.

### Gene ontology (GO) and functional pathway analysis

The web tool "jvenn" was used to visualise the total number of proteins and differentially expressed proteins in each group. Dysregulated proteins (up-regulated and down-regulated) were annotated and categorised as a biological process (BP), cellular component (CC), and molecular function (MF) and Kyoto Encyclopaedia of Genes and Genomes (KEGG) pathway^61^ using Uniprot and the Database for Annotation, Visualization, and Integrated Discovery (DAVID) Bioinformatics Resources v6.8 (Laboratory of Human Retrovirology and Immunoinformatics, USA)^62^. Gene ontology (GO) bubble charts for the top 10 dysregulated BPs, MFs, and CCs were displayed using the R package "ggplot2". DAVID^63^, an online annotation software, was used to find significantly differentially expressed proteins with a p-value cut-off of 0.05, and R package “clusterProfiler” was used plot the enriched pathways^64^. ClueGo (Version 2.5.4, Integrative Cancer Immunology, Jerome Galon) and Cluepedia (Version 1.5.4) plugins in the open-source Cytoscape (Version3.7.1, National Institute of General Medical Sciences (NIGMS), USA) platform were used to perform interaction network analysis of combined GO categories and pathway analysis among the proteins^65^. The interaction between proteins was shown using STRING v11.5 (Search Tool for the Retrieval of Interacting Genes/Proteins; https://string-db.org/), an online tool^66^. The heatmap was created using the R package "gplots", which uses 'heatmap.2' function and protein abundance values to plot the heatmap. In the heatmap, we plotted each of the top 15 up- and down-regulated proteins.

## Supplementary Information

Supplementary Figures.

Supplementary Information 1.

Supplementary Information 2.

Supplementary Information 3.

## Data Availability

All data generated or analysed during this study are included in this published article (and its Supplementary Information files).
