# Supplementary material for: Establishment of a repertoire of fertility associated sperm proteins and their differential abundance in buffalo bulls (Bubalus bubalis) with contrasting fertility
Source: Sci Rep. 2023 Feb 8;13:2272. doi: 10.1038/s41598-023-29529-5 (PMC9908891; doi:10.1038/s41598-023-29529-5)
Supplement: Supplementary file 1 — Supplementary Figures. [file 41598_2023_29529_MOESM1_ESM.docx]

**SUPPLEMENTARY FIGURES**


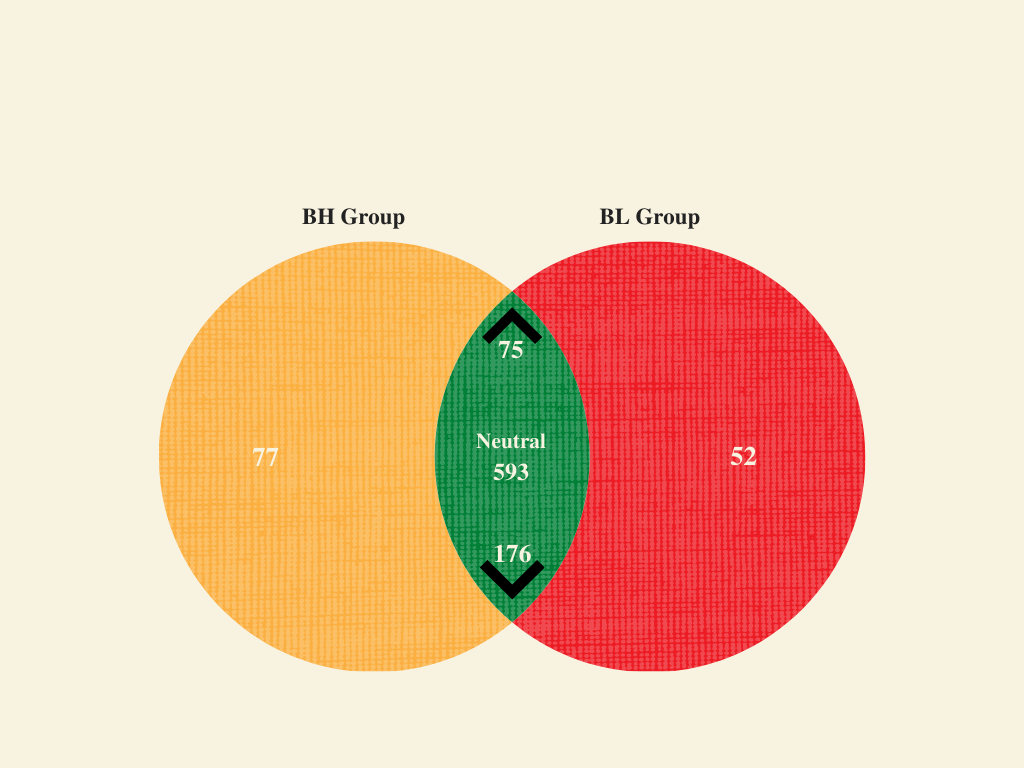


**Supplementary Fig 1.** Venn diagram indicating the number of proteins detected in high- (BH) and low- (BL) fertile buffalo bull spermatozoa.


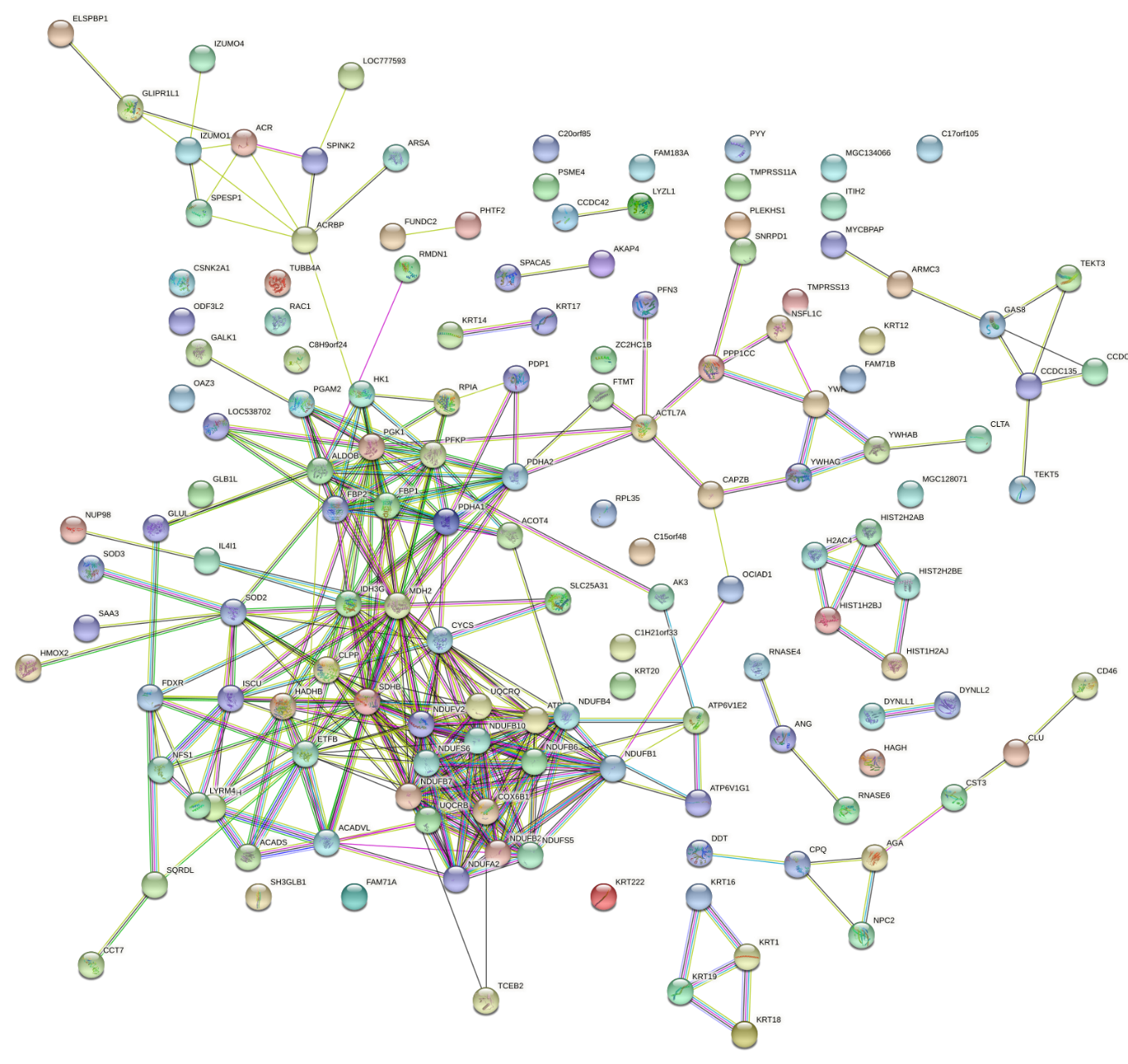


**Supplementary Fig 2.** Protein-protein interaction network of dysregulated sperm proteins between high- and low-fertile buffalo bulls. Network was created using STRING interaction software Version 11.5 (https://string-db.org/cgi/input?sessionId=b6VdP5zN42xn&input_page_active_form=multiple_identifiers).
